# Circular dichroism spectroscopy reveals multiple phytochrome photoproducts in equilibrium

**DOI:** 10.1007/s43630-025-00763-2

**Published:** 2025-07-18

**Authors:** Nathan C. Rockwell, J. Clark Lagarias

**Affiliations:** Department of Molecular and Cell Biology, University of California at Davis, One Shields Avenue, 31 Briggs Hall, Davis, CA 95616 USA

## Abstract

Phytochromes are a widespread family of red/far-red photoreceptors including master regulators of plant growth and development. Phytochromes use 15,16–photoisomerization of linear tetrapyrrole (bilin) chromophores to toggle between a *15Z* red-absorbing dark-adapted state (P_r_) and a *15E* far-red-absorbing photoproduct (P_fr_). The bilin is bound within a conserved, N-terminal PAS-GAF-PHY photosensor tridomain and is covalently attached to a conserved Cys residue, but the mechanism(s) permitting detection of far-red light are not well understood. Plant and cyanobacterial phytochromes exhibit complex P_fr_ CD spectra that are also not well explained. In this work, we use the model cyanobacterial phytochrome Cph1 from *Synechocystis* sp. PCC 6803 to examine the basis for this complex CD spectrum. We employ truncations with and without the PHY domain (N514 and N322) as well as a panel of variants with point substitutions in N514. We identify two classes of photoconversion: type 1 produces P_fr_, whereas type 2 produces a blue-shifted alternative photoproduct (P_ALT_) with a distinct CD spectrum and with properties similar to those of the previously observed Meta-R_C_ intermediate. Both type 1 and type 2 variants exhibit efficient photoisomerization, indicating that type 2 variants are specifically deficient in spectral tuning of the 15E photoproduct. Subtle differences within type 1 variants can be ascribed to the presence of varying amounts of P_ALT_. We show that P_fr_ formation can proceed at pH 6 in Type 2 cases, whereas even wild-type N514 is unable to form P_fr_ at pH 9. We, thus, demonstrate that the photoproduct of Cph1 contains two *15E* species in pH-dependent equilibrium, shedding new light on the P_fr_ state.

**Supplementary Information:**

The online version contains supplementary material available at 10.1007/s43630-025-00763-2.

## Introduction

Living organisms use the ambient light environment for many purposes. For example, animals can use visual cues for foraging, for social displays, or for avoiding predation. Photosynthetic organisms use light for carbon fixation, but they also employ a range of photoreceptors to optimize this process. For example, plant phytochromes measure the ratio of red to far-red light (the ratio of light intensity at ca. 660–670 nm to that at ca. 720–730 nm) using photoisomerization of a linear tetrapyrrole (bilin) chromophore. This ratio serves as a proxy to detect competition for photosynthetically active red light from taller plants, allowing the plant to regulate growth and developmental programs in response to the light environment (photomorphogenesis) [1–3]. Phytochromes are also found in fungi, diverse eukaryotic algae, and a range of bacteria [4]. Cyanobacteria have a particularly diverse palette of phytochromes, including examples that can respond to violet light [5–8] as well as conventional red/far-red phytochromes such as the well-studied model protein Cph1 from *Synechocystis* sp. PCC 6803 [5, 9–22]. Phytochromes have also attracted considerable attention as optogenetic and biotechnological research tools. Engineering the behavior of plant phytochromes not only promises to improve crop yield by controlling shade avoidance responses [23], but also promises to allow applications in mammalian tissues that are more transparent at red/far-red wavelengths. [24–35]

A wealth of spectroscopic and structural information has been obtained for phytochromes such as Cph1. Such photoreceptors share an N-terminal photosensory core module (PCM) consisting of PAS, GAF, and PHY domains (Fig. S1A) in a unique knotted architecture [15, 36–39]. The bilin chromophore is covalently attached to a conserved Cys residue and lies within a conserved pocket in the GAF domain. Most phytochromes have two photostates: a red-absorbing dark-adapted state (P_r_) and a metastable far-red-absorbing photoproduct (P_fr_) that can revert to P_r_ on a timescale of hours in the absence of light (dark reversion). Photoconversion between P_r_ and P_fr_ is accomplished via initial photoisomerization of the 15,16-double bond of the bilin chromophore, which can be biliverdin IXα (BV), phytochromobilin (PΦB), or phycocyanobilin (PCB) [1, 4, 37, 40–42]. This photochemical reaction initially generates a primary photoproduct which then evolves via several intermediates to generate the final photostate. Forward photoconversion is thought to proceed through a deprotonated Meta-Rc intermediate [11, 43], and structural changes in the chromophore-binding pocket are propagated through the protein to modulate the activity of effector domains that interface with cellular signal transduction pathways [44–46].

Despite these extensive studies, the molecular basis for far-red absorption by P_fr_ photoproducts of phytobilin-based plant and cyanobacterial phytochromes remains opaque. Protonated *15E* PCB has an intrinsic peak absorption at ca. 610 nm, [5, 47–55] in the orange region of the visible spectrum, yet in native, wild-type Cph1, this same chromophore is able to detect far-red light at 720 nm [10, 12, 16]. The mechanistic basis for this tuning effect is not fully understood. Phytochromes using BV generate a distinct P_fr_ state relative to those of Cph1 or plant phytochromes as judged by circular dichroism (CD) spectroscopy, by unequal effects of equivalent substitutions in conserved residues, and by the effects of incorporating non-physiological chromophores such as bilin amides [13, 16, 56, 57]. By contrast, studies using crystallography and NMR spectroscopy confirm that photoconversion proceeds from a C5–*Z,syn* C10–*Z,syn* C15–*Z,anti* geometry to a C5–*Z,syn* C10–*Z,syn* C15-*E,anti* geometry for both BV- and PCB/PΦB-based phytochromes [15, 36–38, 40, 41, 58–60]. The CD signatures of all P_r_ states are conserved: the red-absorbing S_0_–S_1_ transition is associated with negative CD, and the near-UV S_0_–S_2_ (Soret) transition is associated with a single, positive band [16]. These signals have been interpreted as arising from an α-facial D-ring, which is observed in P_r_ structures [15, 16, 36, 38, 39, 61] and is also consistent with studies on photoisomerization of phycoviolobilin (PVB) in the photoactive biliprotein α-phycoerythrocyanin [62–65]. Bacteriophytochromes such as DrBphP and PaBphP utilize BV and generate a red-shifted P_fr_ state with CD spectra similar to those of P_r_, again consistent with the α-facial D-ring observed in the crystal structure of the P_fr_ state of PaBphP from *Pseudomonas aeruginosa*. [16, 37]

Interpretation of the equivalent spectra for Cph1 or plant phytochromes is less straightforward. In these cases, P_fr_ exhibits an anomalous CD spectrum [11, 16, 66]: the far-red-absorbing S_0_–S_1_ transition is associated with positive CD, whereas the Soret (S_0_–S_2_) region exhibits complex CD with a positive band at longer wavelengths, a negative region, and another positive region in the far-red to near-infrared (Fig. S1B, C). The positive CD seen in the S_0_–S_1_ transition has been proposed to arise from a β-facial D-ring, consistent with magic angle spinning (MAS)-NMR studies on Cph1 and plant phytochrome truncations [16, 40, 60]. Such P_fr_ states can also exhibit variable negative CD signal in the red region. CD signals from full-length and truncated Cph1 are nearly indistinguishable throughout the near-UV to the near-IR [11, 16]. Negative CD in the red region is consistent with the presence of residual P_r_ at photoequilibrium. However, similar CD signals are also observed in the P_fr_ state of CparGPS1 from the glaucophyte alga *Cyanophora paradoxa*, an unusual phytochrome with a blue/far-red photocycle that lacks a P_r_ dark state [67]. The presence of residual P_r_ is, thus, not sufficient to explain the complex CD spectra of such P_fr_ states in the Soret region. Analysis of CparGPS1 and of phytochromes from prasinophyte algae led to the proposal that the complex CD signatures of P_fr_ arise from a heterogeneous mix of species with opposing CD [67]. However, other interpretations of this behavior are conceivable. For example, vibrational fine structure can also lead to complex CD spectra, as is the case in the spectra of phenylalanine and its derivatives [68]. Computational studies of chlorophylls have provided support for strong vibronic coupling in some tetrapyrroles, such that the distinction between S_0_–S_1_ and S_0_–S_2_ transitions breaks down entirely. [69]

The present study was undertaken to elucidate the biochemical basis for the complex P_fr_ CD spectrum of phytobilin-based phytochromes using Cph1 as a model. Characterization of truncation constructs with or without the PHY domain and of variant proteins with point substitutions in the GAF or PHY domains identifies two types of photoproduct: the true P_fr_ state associated with positive CD, and a blue-shifted alternative photoproduct associated with negative CD. We demonstrate that these two photoproducts are in pH-dependent equilibrium even in the absence of the PHY domain. This work, thus, provides new insight into the complex process of photoconversion in a model phytochrome.

## Results

### The PHY domain of Cph1 is required for Pfr formation but not for photoisomerization

**Fig. 1:**
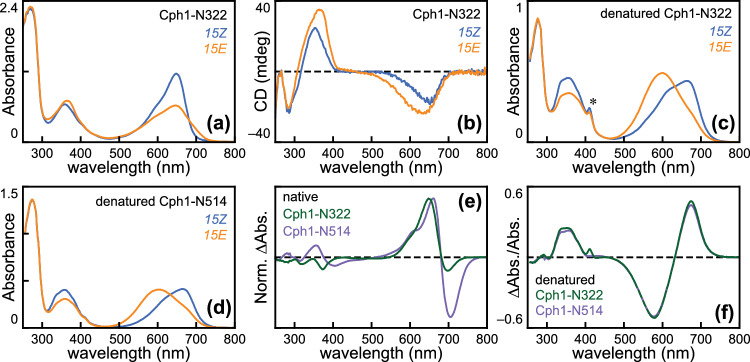
The Cph1 PHY domain is required for P_fr_ formation but not photoisomerization. **a** Absorption spectra are shown for Cph1-N322 in the *15Z* (blue) and *15E* (orange) states. **b** CD spectra are shown for the same sample as in panel (a), using the same color scheme. **c** Absorption spectra are shown for an aliquot of the same sample under acidic denaturing conditions, using the same color scheme. Asterisk, porphyrin contaminant. **d** Absorption spectra are shown for Cph1-N514 under denaturing conditions, using the same color scheme. **e** Normalized photochemical difference spectra are shown as (*15Z* –*15E*) for Cph1-N322 (dark green) and Cph1-N514 (violet) under native conditions. Difference spectra were normalized on the *15Z* peak. (f) Photochemical difference spectra are shown for Cph1-N322 and Cph1-N514 under denaturing conditions, using the same color scheme. Difference spectra were normalized using the peak *15Z* absorbance for each construct, allowing an approximate comparison of relative efficiency of photoisomerization

Previous reports have shown that photoconversion of the N514 construct (with a complete PAS-GAF-PHY PCM) yields a P_fr_ photoproduct that is spectroscopically indistinguishable from that of full-length Cph1, whereas the shorter PAS-GAF truncation (hereafter, N322; Fig. S1A) affords a bleached and blue-shifted photoproduct [10, 13, 16, 22, 70]. Here we replicate these observations (Figs. 1A and S1B; Table 1) and show that the N514 and N322 photoproducts have strikingly different CD spectra (Figs. 1B and S1C; Table 2). Unlike the complex CD observed for the P_fr_ state of N514, the N322 photoproduct exhibited a simple CD spectrum with a single positive band in the Soret region and a single negative band matching the broad red-to-orange absorption peak of the same state. The characteristic inversion of the S_0_–S_1_ band seen in plant phytochromes, in N514, and in full-length Cph1 [11, 16, 66] was, thus, absent in N322. This spectrum was distinct from, albeit overlapping, that of P_r_, and significant regeneration of P_r_ was not observed during acquisition of CD spectra (approximately 3 min).

**Table 1:** Characterization of Cph1 variants using absorption spectroscopy^a^

Cph1 variant	Type	SAR	% res. *15Z*	*15Z* λ_max_	∆Abs λ_max/min_
Wild-type N514	1	0.9	20	362, 662	660; 702
N514 F_178_A	2	0.2	< 10	360, 652	654; 536, 704
N514 F_178_I	2	0.4	< 10	362, 656	658; 538, 710
N514 Y_198_F	1	0.8	n/d	360, 660	658; 704
N514 L_201_A	2	0.2	< 10	362, 644	648; 466, 702
N514 H_202_A	†	0.3	n/d	360, 650	648; 704
N514 Y_203_A	2	0.1	n/d	364, 642	642; 538, none
N514 Y_203_A (IVA)	2	0.6	n/d	362, 646	648; 532, 692
N514 Y_203_F	1	1.1	15–25	362, 660	656; 702
N514 S_206_A	1	0.6	n/d	360, 654	650; 706
N514 D_207_N	2	0.3	< 15	364, 642	656; 542, none
N514 I_208_V	1	0.7	5–15	360, 660	658; 704
N514 I_208_L	1	0.6	20	358, 648	590, 642; 698
N514 M_267_A	†	0.5	n/d	358, 648	648; 700
N514 H_290_A	2	0.7	n/d	360, 650	652; 538, 704
N514 R_441_A	1	0.6	20	363, 656	652; 704
N514 W_450_A	1	0.4	< 15	358, 648	646; 702
N514 S_474_A	2	0.3	< 10	364, 654	662; 540, 750
N514 S_474_C	2	0.6	< 5	364, 662	664; 542, 706
N514 F_475_A	2	0.4	< 10	362, 646	646; 474, 700
N514 W_478_A	2	0.4	< 10	366, 646	650; 510, 700
N514 E_480_A	1	0.5	25–30	360, 654	648; 704
wild-type N322	2	0.5	15–20	358, 648	648; 514, 698

^a^SAR (specific absorbance ratio) is reported for native samples and was calculated as described in the Methods. %*15E* was estimated from denatured spectra as described in Methods (Fig. S2). Peak wavelengths are reported in nm. Assignment to Type 1 or Type 2 was done using difference spectra

† indicates variants which behaved differently in absorption and CD spectroscopy (cf. Table 2). IVA, holoprotein was generated via in vitro assembly with PCB

**Table 2:** Characterization of Cph1 variants using CD spectroscopy^a^

Cph1 variant	Type	*15Z* λ_max_	*15Z* CD/Abs	*15E* λ_max_	*15E* CD/Abs
Wild-type N514	1	350, 660	+ 45, – 15	379, 698	– 7.3, + 22
N514 F_178_I	2	358, 654	+ 37, – 25	368, 602	+ 31, –38
N514 Y_198_F	1	352, 656	+ 30, – 12	388, 704	– 2.9, + 7.8
N514 L_201_A	2	354, 644	+ 31, – 25	368, 636	+ 34, –43
N514 H_202_A	†	354, 650	+ 27, – 22	368, 626	+ 35, –41
N514 Y_203_A	2	356, 636	+ 34, – 31	356, 636	+ 34, –45
N514 Y_203_F	1	354, 660	+ 42, – 16	382, 702	– 17, + 18
N514 S_206_A	1	354, 658	+ 34, – 14	390, 706	– 5.3, + 18
N514 D_207_N	2	364, 634	+ 48, – 38	368, 624	+ 47, –57
N514 I_208_V	1	352, 656	+ 29, – 18	386, 704	– 8.2, + 21
N514 I_208_L	1	352, 652	+ 33, – 15	386, 698	– 13, + 21
N514 M_267_A	†	344, 650	+ 20, – 7.4	360, 646	+ 23, –17
N514 H_290_A	2	352, 648	+ 37, – 19	362, 634	+ 27, –24
N514 R_441_A	1	352, 656	+ 32, – 16	390, 704	– 3.3, + 17
N514 W_450_A	1	350, 656	+ 39, – 16	412, 702	+ 0.2, + 25
N514 S_474_A	2	364, 636	+ 57, – 42	364, 622	+ 59, –62
N514 S_474_C	2	358, 660	+ 44, – 20	362, 620	+ 56, –49
N514 F_475_A	2	352, 644	+ 26, – 14	358, 616	+ 27, –32
N514 W_478_A	2	358, 624	+ 47, – 24	364, 628	+ 56, –53
N514 E_480_A	1	354, 646	+ 32, – 14	376, 698	– 13, + 27
wild-type N322	2	354, 652	+ 39, – 16	364, 634	+ 50, –39

^a^Type assignments were made using the *15E* CD spectra

† indicates cases which were assigned to different types in CD and absorption spectroscopy (cf. Table 1). Peak wavelengths are in nm. Relative rotational strength (CD/Abs) was calculated by dividing the observed strongest CD (highest positive or lowest negative value) by the peak absorbance value for the same band

Examination of the resulting photoproducts using an acid denaturation assay [5, 41, 47, 51, 52, 55, 71] demonstrated that chromophores of both constructs exhibit the same underlying configurational changes upon photoconversion (Fig. 1C, D). To estimate the extent of photoconversion for the N514 and N322 proteins at photoequilibrium under red light, we compared the absorption spectrum of the denatured photoproduct to a panel of calculated reference spectra with varying levels of *15E* content [54]. This analysis found little to no difference between them within the resolution of this approach (Fig. S2A-C; Table 1). Consistent with this, the difference spectra for photoconversion of these two constructs under denaturing conditions were nearly identical, despite the clear differences for native proteins (Fig. 1E, F). Denatured difference spectra (Fig. 1F) were normalized by chromophore absorbance (∆A/A), indicating that roughly equivalent amounts of photoisomerization occurred in the two constructs because roughly equivalent amounts of *15E* bilin were trapped by denaturation. We conclude that the absence of the PHY domain does not block 15,16-photoisomerization but does prevent subsequent P_fr_ formation, indicating that Cph1 can efficiently form an alternative, blue-shifted *15E* photoproduct in the absence of the PHY domain. This analysis, thus, implies that the *15E* N322 photoproduct must be less photoactive than the *15Z* dark-adapted state under red light, even though both species can absorb it (Fig. 1A, B), such that this light condition fortuitously drives forward photoconversion to the same extent as N514.

### Variant Cph1 proteins identify two types of photoconversion

**Fig. 2:**
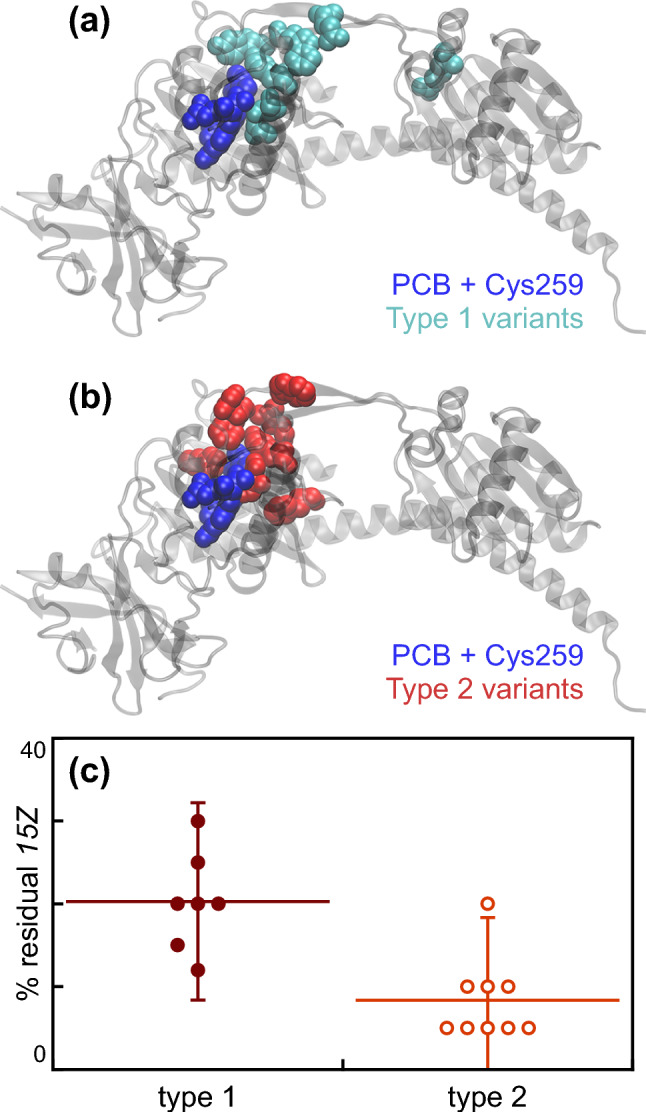
Analysis of variant Cph1-N514 proteins exhibiting different types of photoconversion. **a** The crystal structure of the PAS-GAF-PHY N-terminal fragment of Cph1 (PDB accession 2VEA) in its *15Z* dark state is shown [15]. Substitution of the amino acids highlighted in cyan resulted in variants exhibiting Type 1 photoconversion with substantial P_fr_ formation. The PCB chromophore is highlighted in blue. **b** The same structure is shown, with PCB again highlighted in blue. Substitution of the amino acids highlighted in red resulted in variants exhibiting Type 2 photoconversion, mimicking Cph1-N322. **c** The residual *15Z* population present in the photoproducts of Cph1 variants was estimated using the denaturation assay (Fig. S2) and is plotted for selected Type 1 and Type 2 cases. Error bars are set at two standard deviations from the mean

We extended these observations by characterizing a panel of variant N514 proteins containing substitutions in conserved residues and/or residues structurally proximal to the chromophore (Fig. 2 and Table 1). We targeted a number of residues that were highly conserved in Cph1 and other PCB-utilizing cyanobacterial phytochromes [8] and/or close to the chromophore in the wild-type Cph1 crystal structure [15]. We omitted substitutions at Tyr176, His260, and Tyr263 due to previous studies of such variants in Cph1 [13, 14, 17]. Although substitutions at Tyr176 can block photoisomerization, thereby resulting in bright red fluorescence [12, 13], the small amount of photoproduct that is formed is spectrally similar to that seen in wild-type N514. Substitutions at His260 can result in loss of chromophorylation, but the H260Q variant again seems to produce a relatively normal P_fr_ state under standard conditions [14]. Substitutions at Tyr263 have more variable effects; Y263F exhibits a largely normal red/far-red photocycle under static illumination, whereas Y263S and Y263H exhibit more complex behavior in CD spectroscopy [17]. Most variant proteins examined in the current work exhibited absorption spectra broadly similar either to those of wild-type N514 (hereafter, Type 1 variants: Fig. 2A and Tables 1 and 2) or to those of N322 (Type 2: Fig. 2B and Tables 1 and 2). The acid denaturation assay demonstrated that the amount of residual *15Z* chromophore after illumination with red light was not reduced in Type 2 variants (Fig. 2C), confirming that such variants photoisomerize as effectively as Type 1 variants but fail to form the far-red-absorbing Pfr state. This analysis is consistent with apparently poor reverse photoconversion in Type 2 variants under red light, as we also inferred for N322. Characterization of variant proteins using CD spectroscopy (Table 2) demonstrated that Type 1 photoconversion produces “true P_fr_” with far-red absorption and positive long-wavelength CD, whereas Type 2 photoconversion instead yields a broad, blue-shifted *15E* species with negative CD observed for the S_0_–S_1_ transition and simple positive CD observed for the Soret transition. The Type 2 photoproducts of these variant proteins, thus, are similar to the alternative photoproduct observed in N322. We designate this species as P_ALT_.

**Fig. 3:**
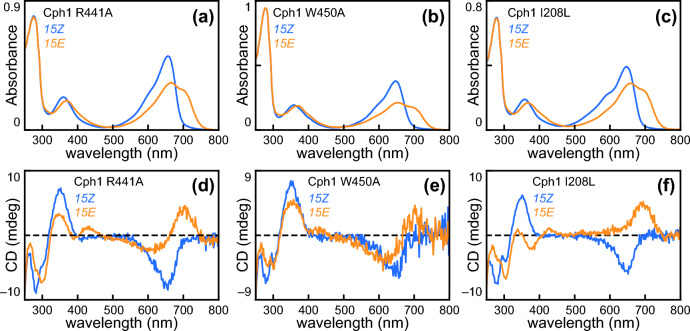
Characterization of representative Type 1 Cph1-N514 variants. **a** Absorption spectra are shown for the R441A variant, using the color scheme of Fig. 1a. **b** Absorption spectra are shown for the W450A variant, using the same scheme. **c** Absorption spectra are shown for the I208L variant, using the same scheme. **d** CD spectra are shown for the R441A variant, using the same scheme. **e** CD spectra are shown for the W450A variant, using the same scheme. (f) CD spectra are shown for the I208L variant, using the same scheme

**Fig. 4:**
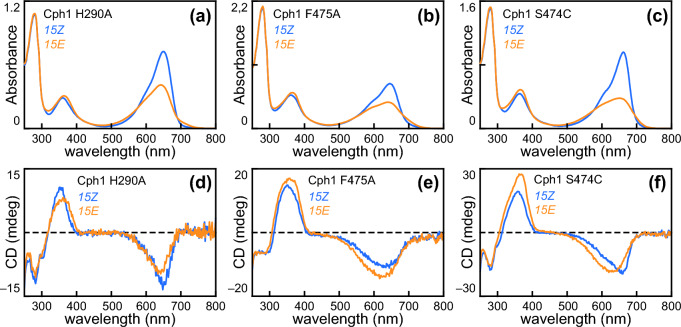
Characterization of representative Type 2 Cph1-N514 variants. **a** Absorption spectra are shown for the H290A variant, using the color scheme of Fig. 1a. **b** Absorption spectra are shown for the F475A variant, using the same scheme. **c** Absorption spectra are shown for the S474C variant, using the same scheme. **d** CD spectra are shown for the H290 variant, using the same scheme. **e** CD spectra are shown for the F475A variant, using the same scheme. **f** CD spectra are shown for the S474C variant, using the same scheme

We observed subtle variation within the two classes of photoproducts. The Type 1 R441A and W450A variants both contain substitutions in conserved residues in the PHY domain, whereas the I208L variant targets the conserved isoleucine of the so-called DIP motif [72, 73]. All three exhibit substantial P_fr_ formation, with I208L Cph1 also showing effects on P_r_ (Fig. 3A–C). These three variants also exhibit differences in CD spectroscopy. The R441A and W450A variants exhibited a clear increase in negative CD at ca. 500–600 nm upon photoconversion to P_fr_ (Fig. 3D, E), whereas the I208L variant exhibits no negative CD at all in this region of the P_fr_ spectrum (Fig. 3F). Signals in this region of the CD spectrum, thus, did not correlate with the amount of residual P_r_ (Fig. 3D–F and Table 1). All three of these variants exhibited varying amounts of positive CD in the region associated with the far-red absorption peak. Other subtle differences were observed in the P_fr_ CD spectra, with notable variation in the positive signals at ca. 350 nm and in the negative signals at ca. 380 nm. By contrast, Type 2 variants showed little to no increase in far-red absorption, as shown by the H290A, F475A, and S474C variants (Fig. 4A–C). His290 forms a hydrogen bond to the chromophore D-ring in the P_r_ state, so the slight blue shift of the P_r_ state of the H290A variant was not unexpected (Fig. 4A and Table 1).

The F475A and S474C variants targeted conserved residues in the PRXSF ‘tongue’ motif that is critical for transmitting light-induced structural changes occurring within the bilin-binding pocket to the rest of the molecule [44, 73–76]. F475A is a less conservative substitution than S474C; it is again associated with a blue-shifted P_r_ spectrum that stands in contrast to the relatively normal P_r_ spectrum of S474C (Fig. 4B, C; Table 1). Photoconversion of S474C also appeared more complete than that of H290A and F475A as judged by depletion of P_r_ absorption (Fig. 4A–C). Remarkably, acid denaturation demonstrated that photoisomerization of this variant to *15E* under red light was effectively complete within the resolution of this assay (Fig. S2D and Table 1). This implies that the photochemical quantum yield for photoisomerization of P_ALT_ must be negligible for S474C, such that a photoequilibrium is not formed. Surprisingly, however, this variant could be restored to the *15Z* P_r_ state via illumination with far-red light, implicating the presence of a very small amount of P_fr_ in equilibrium with P_ALT_. The H290A, S474C, and F475A variants also exhibited subtle differences in CD spectroscopy (Fig. 4D–F). The photoproduct of H290A Cph1 exhibited a narrower peak in the red region of the spectrum than that of F475A (Fig. 4D, E). S474C again exhibited a broad CD peak associated with P_ALT_ (Fig. 4F), in this case with a slight but clear blue shift of the S_0_–S_1_ band upon photoconversion to the *15E* state.

**Fig. 5:**
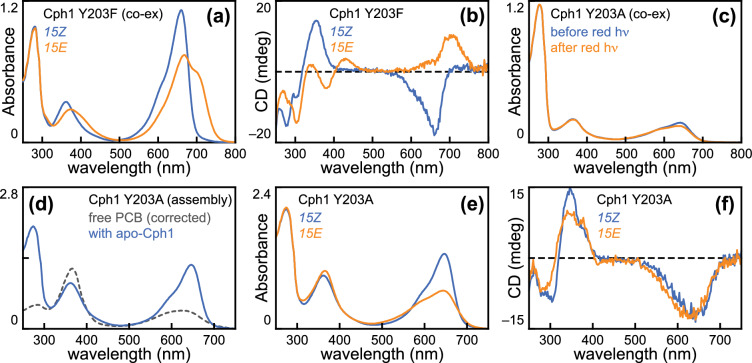
Characterization of substitutions for Tyr203 in Cph1. **a** Absorption spectra are shown for the Y203F variant, using the color scheme of Fig. 1a. **b** CD spectra are shown for the Y203F variant, using the same scheme. **c** Absorption spectra are shown for the Y203A variant after co-expression with PCB biosynthesis enzymes under identical conditions to the Y203F variant. **d** Absorption spectra are shown for in vitro assembly of Y203A apoprotein with free PCB (concentration-corrected free PCB, black dashed line; resulting *15Z* state, blue). **e** Absorption spectra are shown for photoconversion of the Y203A variant after in vitro assembly, using the color scheme of Fig. 1a. **f** CD spectra are shown for the same sample as in panel **e**

Substitutions for the conserved Tyr203 gave rise to both Type 1 and Type 2 variants (Fig. 5), reminiscent of the variable effects seen in variants having substitutions at Tyr263 [17]. The Y203F variant clearly exhibited Type 1 photoconversion (Fig. 5A, B), with a P_fr_ CD spectrum similar to that of the I208L variant (Fig. 3F). Characterization of the Y203A variant prepared using the same co-expression and purification protocol resulted in a poorly photoactive preparation resembling Type 2 variants (Fig. 5C) that was shown to contain large amounts of *15E* bilin in the denaturation assay (CD parameters are reported in Table 2). We were unable to regenerate the *15Z* state with this preparation, again consistent with a low quantum yield for reverse conversion of P_ALT_. We, therefore, purified this variant as an apoprotein for in vitro assembly with PCB (see Methods). This procedure allowed generation of a P_r_ state (Fig. 5D), and photoconversion of this material under red light gave rise to a Type 2 photoproduct (Fig. 5E, F) similar to that seen in the holoprotein preparation. These experiments, thus, demonstrate that Cph1 variants in the context of the PHY domain can exhibit either Type 1 photoconversion, leading to formation of true P_fr_, or Type 2 photoconversion, leading to the alternative photoproduct P_ALT_ also formed in the absence of the PHY domain. Comparison of the peak wavelengths for the two possible photoproducts was ambiguous using absorption spectroscopy, because of the spectral overlap between P_r_ and P_ALT_. However, analysis of the observed peak wavelengths in CD spectroscopy (Fig. S3A) demonstrates that both the S_0_–S_1_ and S_0_–S_2_ transitions are blue-shifted in P_ALT_, with the failure to form true P_fr_ resulting in a blue shift of over 50 nm.

### Type 1 variants also form Type 2 photoproducts

**Fig. 6:**
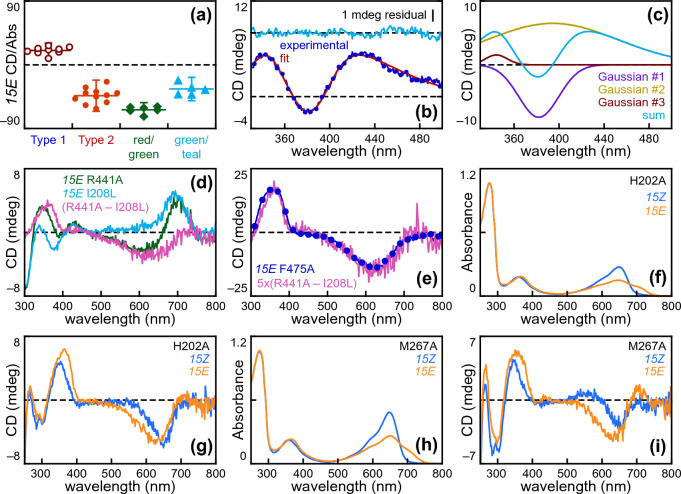
Signals from the Type 2 photoproduct are present in the Pfr CD spectra of Type 1 variants. **a** Relative rotational strength (observed CD divided by peak absorption) is plotted for the *15E* states of Type 1 Cph1 variants (open brick red circles), Type 2 variants (filled dark orange circles), red/green CBCRs (filled green diamonds), [100] and green/teal CBCRs (filled cyan triangles). Error bars are shown at two standard deviations. **b** The Soret window of the Cph1-N514 Pfr CD spectrum (blue line and filled circles) was fit to a linear combination of three Gaussian functions (red line) as described in the Methods. The residual for the fit (cyan trace) is also shown. **c** The components of the Gaussian fit are shown (purple, bronze, and brick red), along with the sum (cyan). **d** *15E* CD spectra of the R441A and I208L variants (dark green and cyan, respectively) are shown, along with the (I208L – R441A) difference spectrum (coral). **e** The difference spectrum from panel (d) (coral) was multiplied by five for comparison to the *15E* CD spectrum of the F475A variant (blue line and filled circles). **f** Absorption spectra are shown for the H202A variant, using the color scheme of Fig. 1a. **g** CD spectra are shown for the H202A variant, using the same color scheme. **h** Absorption spectra are shown for the M267A variant, using the same color scheme. **i** CD spectra are shown for the M267A variant, using the same color scheme

Type 1 variants exhibited differences in the Soret region of the P_fr_ CD spectrum (Fig. 3D, F); for example, the negative peak at ca. 380 nm was much more pronounced for the I208L variant than for the W450A and R441A variants, whereas the converse held true for negative CD associated with the long-wavelength S_0_–S_1_ transition. Such variation is consistent with our earlier proposal that the P_fr_ CD spectrum might arise due to contributions from two species with opposed sign [67], because a varying ratio of two opposed populations might yield variable internal cancelation. Such internal cancelation might result in weaker relative rotational strength for S_0_–S_1_ transitions (CD/Abs, calculated as observed CD in mdeg per unit Absorbance) in Type 1 variants than in Type 2 variants. We, therefore, calculated CD/Abs ratios for Type 1 and Type 2 variants (Fig. 6A). From this comparison, it is clear that Type 1 variants exhibited weaker relative rotational strength than Type 2 variants. The negative CD/Abs of Type 2 variants was lower than that seen for red/green cyanobacteriochromes (CBCRs), a group of distant phytochrome relatives known to form green-absorbing photoproducts with significant ring tilt at both the A- and D-rings [77, 78], and similar to that of green/teal CBCRs, which form a similar twisted photoproduct but which incorporate phycoviolobilin and hence have no CD or absorption contribution from the A-ring [52, 79]. The Type 2 photoproduct P_ALT_, thus, has relative rotation that is stronger than that observed for Type 1 but lower than that expected for a highly twisted PCB adduct.

We next fit the Soret region for a representative P_fr_ CD spectrum to a linear combination of Gaussian functions (Fig. 6B, C), mimicking our earlier analysis of algal phytochromes [67]. Good results were obtained with three Gaussians (Fig. 6B). Analysis of these functions revealed three distinct components (Fig. 6C): a negative component at ca. 380 nm, a broad positive component at longer wavelengths, and a weak positive component at shorter wavelengths similar to the observed Soret CD peak wavelengths of the Type 2 F475A and Y203A variants (Figs. 4D and 5F; Table 2). These components, thus, are good matches for the observed variation in the Soret regions of P_fr_ CD spectra for Type 1 variants. The similarity between the short-wavelength, positive Gaussian and the observed peaks of Type 2 variants seemed consistent with the presence of varying amounts of the Type 2 photoproduct P_ALT_ in Type 1 cases. As a first evaluation of this hypothesis, we compared the P_fr_ CD spectra of the R441A and I208L variants (Fig. 6D). The (R441A–I208L) difference CD spectrum exhibited the features of a Type 2 photoproduct CD spectrum: negative CD in the S_0_–S_1_ region and simple positive CD in the Soret region. This difference spectrum was also very similar to the observed CD spectrum of P_ALT_ for the Type 2 variant F475A after scaling (Fig. 6E), indicating that the observed differences between these two Type 1 variants are indeed consistent with the presence of a variable amount of P_ALT_ in both of them.

Further support for the presence of both photoproducts in Type 1 variants came from characterization of the H202A and M267A variants (Fig. 6F–I; Tables 1 and 2). In both cases, absorption spectroscopy clearly showed P_fr_ formation, which would support assignment of these variants as Type 1. However, CD spectra showed the formation of a negative population in the orange to red region of the spectrum upon photoconversion and of a simple, positive Soret band in the photoproduct, both of which are hallmarks of Type 2 photoconversion. Hence, these two variants must make both photoproducts upon photoconversion of P_r_. Moreover, reverse photoconversion of the M267A variant could be effected using far-red-light with simultaneous loss of the P_ALT_ CD signals. Depletion of P_ALT_ by illumination of true Pfr implies that the two *15E* species present in the M267A variant are in equilibrium with each other. These experiments, thus, demonstrate that variants exhibiting Type 1 photoconversion also produce varying amounts of the Type 2 photoproduct and imply that these two species are in equilibrium.

### Cph1 photoproducts are in a pH-dependent equilibrium

**Fig. 7:**
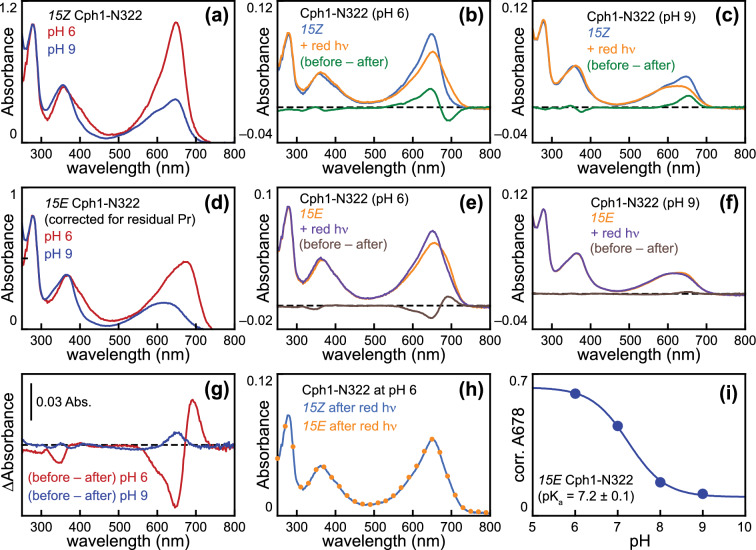
Formation of both type 1 and type 2 photoproducts in Cph1-N322. **a** Absorption spectra are shown for *15Z* Cph1-N322 after tenfold dilution into buffers at pH 6 (red) or pH 9 (blue). **b** Absorption spectra are shown for *15Z* Cph1-N322 at pH 6 before (blue) and after (orange) illumination with red light, along with the resulting (*15Z*–*15E*) difference spectrum (green). **c** Absorption spectra are shown for *15Z* Cph1-N322 at pH 9 before (blue) and after (orange) illumination with red light, along with the resulting (before–after) difference spectrum (green). **d** Absorption spectra are shown for *15E* Cph1-N322 after tenfold dilution into buffers at pH 6 (red) or pH 9 (blue). **e** Absorption spectra are shown for *15E* Cph1-N322 at pH 6 before (orange) and after (purple) illumination with red light, along with the resulting (before–after) difference spectrum (brown). **f** Absorption spectra are shown for *15E* Cph1-N322 at pH 9 before (orange) and after (purple) illumination with red light, along with the resulting (before–after) difference spectrum (brown). **g** The (before–after) difference spectra for *15E* Cph1-N322 at pH 6 (red, panel (e)) and pH 9 (blue, panel (f)) are shown in detail. **h** The endpoints obtained after illumination of Cph1-N322 at pH 6 in the *15Z* (blue line) or *15E* (orange filled circles) states are shown. **i** Absorption spectra for *15E* Cph1-N322 were corrected for buffer effects and residual *15Z* species, and absorbance at 678 nm from the resulting spectra were plotted versus pH to estimate the pK_a_ value of the *15E* bilin in Cph1-N322. Data were fit to a model with a single titrating moiety as described.^54,81^

Relative to P_fr_, P_ALT_ is blue-shifted (Fig. S3A) but has a rather broad absorption. Type 2 variants typically exhibit substantial bleach of the P_r_ state with less obvious photoproduct formation, so the *15E* photoproduct in such cases could have a lower extinction coefficient. These properties are reminiscent of those reported for deprotonated populations observed during detailed characterization of the Cph1 P_r_ state [21, 22, 80]. A deprotonated intermediate is known to form after photoisomerization during forward photoconversion of P_r_ to P_fr_, [21, 43] underscoring the possibility that the Type 2 photoproduct might also be deprotonated. We, therefore, tested this hypothesis. We began with N322, which cannot normally form P_fr_ [22, 70]. N322 Cph1 in either photostate was diluted into 10 volumes of buffer solution at different pH values, examined by absorption spectroscopy, and then illuminated with pulses from a handheld red laser pointer until an endpoint was reached [81]. Consistent with previous studies [21, 22, 80], the *15Z* photostate exhibited bleaching after dilution at high pH (Fig. 7A). Illumination of the *15Z* state revealed a striking pH effect: at pH 6, the N322 construct was able to form P_fr_ (Fig. 7B). This was not true at pH 9 (Fig. 7C). Consistent with this, the *15E* photostate exhibited higher far-red absorption at pH 6 than at pH 9 (Fig. 7D). Red illumination of the *15E* photostate at pH 6 resulted in the surprising formation of P_r_ rather than P_fr_ (Fig. 7E), with an inverted Type 1 difference spectrum, whereas illumination at pH 9 instead resulted in a Type 2 difference spectrum (Fig. 7E–G). The apparent endpoint for photoconversion at pH 6 was the same regardless of the starting photostate (Fig. 7H), consistent with establishment of a different photoequilibrium at pH 6 than at the starting pH of the protein preparation (pH 7.8; see Discussion). Using the estimated *15Z* content under these conditions, we subtracted scaled *15Z* spectra from the *15E* spectra to estimate a pK_a_ value for the *15E* state. The apparent transition was well described by a model assuming a single titrating moiety, with an estimated pK_a_ of approximately 7.2 (Fig. 7I).

**Fig. 8:**
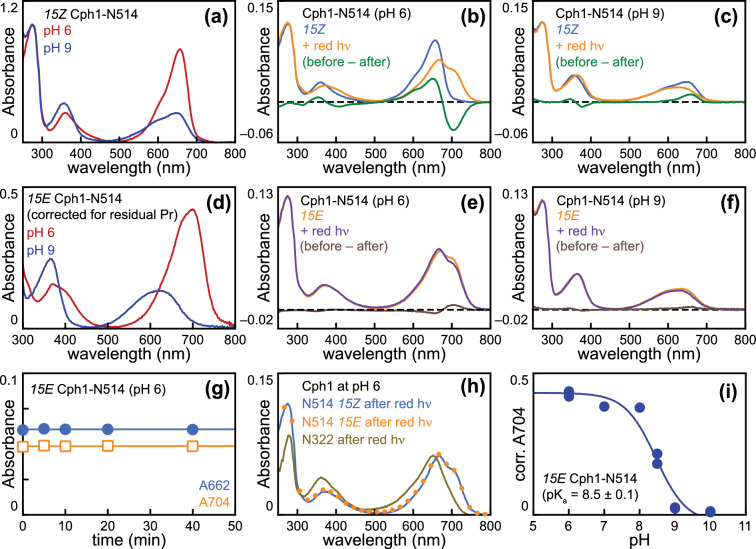
Formation of both type 1 and type 2 photoproducts in Cph1-N514. **a** Absorption spectra are shown for *15Z* Cph1-N514 after tenfold dilution into buffers at pH 6 (red) or pH 9 (blue). **b** Absorption spectra are shown for *15Z* Cph1-N514 at pH 6 before (blue) and after (orange) illumination with red light, along with the resulting (*15Z*–*15E*) difference spectrum (green). **c** Absorption spectra are shown for *15Z* Cph1-N514 at pH 9 before (blue) and after (orange) illumination with red light, along with the resulting (before–after) difference spectrum (green). **d** Absorption spectra are shown for *15E* Cph1-N514 after tenfold dilution into buffers at pH 6 (red) or pH 9 (blue). **e** Absorption spectra are shown for *15E* Cph1-N514 at pH 6 before (orange) and after (purple) illumination with red light, along with the resulting (before – after) difference spectrum (brown). **f** Absorption spectra are shown for *15E* Cph1-N514 at pH 9 before (orange) and after (purple) illumination with red light, along with the resulting (before–after) difference spectrum (brown). **g** Dark reversion of *15E* Cph1-N514 at pH 6 was assayed using absorbance at 664 nm (blue filled circles) and 704 nm (orange open squares). (h) The endpoints obtained after illumination of Cph1-N514 at pH 6 in the *15Z* (blue line) or *15E* (orange filled circles) are shown, with the equivalent spectrum for Cph1-N322 (bronze line) shown for comparison. **i** Absorption spectra for *15E* Cph1-N514 were corrected for buffer effects and residual *15Z* species, and absorbance at 704 nm from the resulting spectra were plotted versus pH to estimate the pK_a_ value of the *15E* bilin in Cph1-N514. Data were fit as in Fig. 7i

We next performed similar experiments with N514 Cph1. The *15Z* state again exhibited bleaching at high pH (Fig. 8A), with increased absorption at ca. 550 nm as expected for a deprotonated bilin sub-population [21, 22, 54, 80, 81]. P_fr_ formation under red illumination was robust at pH 6 but was largely absent at pH 9 (Fig. 8B, C). The *15E* state exhibited higher far-red absorption at pH 6 than at pH 9, and illumination at pH 6 again resulted in formation of P_r_ rather than P_fr_ (Fig. 8D, E). Illumination at pH 9 again resulted in Type 2 photoconversion (Fig. 8F). Control incubations confirmed that dark reversion of the P_fr_ state at pH 6 was slow on the timescale of these experiments (under 10 min after dilution; Fig. 8G), indicating that the reversed photochemical difference spectrum under these conditions was due to (re)establishment of a photoequilibrium rather than due to thermal instability of the *15E* state. Consistent with this interpretation, the endpoint achieved upon illumination at pH 6 was the same regardless of starting photostate for N514 but was distinct from that obtained with N322 at pH 6 (Fig. 8H). The estimated pK_a_ for *15E* N514 was 8.5, higher than that estimated for N322 (Fig. 8I) and comparable to a previously reported value of 9.1 [21]. These experiments, thus, reveal one function of the PHY domain in spectral tuning of P_fr_: it affects a pH-dependent photoproduct equilibrium between true P_fr_ and P_ALT_ by modulating the pK_a_ of the photoproduct. This result is, thus, somewhat different from the behavior of the H260Q variant examined in the presence of the PHY domain, in which the apparent pK_a_ is shifted in both photostates. [14]

**Fig. 9:**
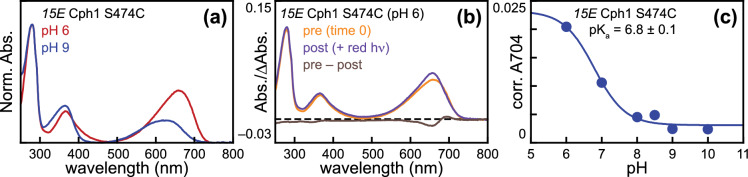
Formation of both type 1 and type 2 photoproducts in the S474C variant. **a** Absorption spectra are shown for the S474C variant of Cph1-N514 in the *15E* state after tenfold dilution into buffers at pH 6 (red) or pH 9 (blue). **b** Absorption spectra are shown for the S474C variant in the *15E* state at pH 6 before (orange) and after (purple) illumination with red light, along with the resulting (before–after) difference spectrum (brown). **c** Absorption spectra for *15E* S474C Cph1 were corrected for buffer effects, and absorbance at 704 nm from the resulting spectra was plotted versus pH to estimate the pK_a_ value of the *15E* bilin in Cph1-N514. Data were fit as in Fig. 7i. Correction for residual *15Z* species was not necessary, due to the efficient photoconversion of this variant (Fig. S2D and Table 1)

We next examined the S474C variant, which exhibits approximately quantitative formation of the *15E* state at pH 7.8 (Fig. S2D). This behavior allowed us to perform an equivalent experiment without the need to subtract residual *15Z* signals. The results were similar to those obtained with N322, including the formation of P_fr_ at pH 6 and the inverted photochemistry observed upon illumination of P_fr_ at pH 6 (Fig. 9A, B). The estimated pK_a_ in S474C was shifted to an even lower value of approximately 6.8 (Fig. 9C), a value low enough to be relatively poorly defined due to loss of protein stability at pH < 6. Comparison of peak S_0_–S_1_ wavelengths at pH 6 and pH 9 demonstrated that the P_r_ state was largely insensitive to pH in this range, consistent with previous studies [21, 80], whereas all three photoproducts were blue-shifted relative to P_r_ at pH 9 (Fig. S3B). We also examined the ratio of intensities for the S_0_–S_1_ and S_0_–S_2_ transitions (S1:S2 ratio, Fig. S3C), which can be sensitive to chromophore configuration and protonation [13, 54, 57]. Both *15Z* and *15E* species exhibited pH-sensitive S1:S2 ratios (Fig. S3D). The effect on the *15Z* state is consistent with the known properties of the Y176H variant and with the known pH-dependent equilibrium between P_r_ populations (see Discussion) [12, 13, 19, 21, 40, 80]. Notably, the protonated P_fr_ species formed by the Type 1 N514 had a higher S1:S2 ratio and longer peak wavelength than those of the Type 2 N322 and S474C (Fig. S3B and D), indicating that these variants are having additional effects on spectral tuning. These experiments demonstrate a pH-dependent equilibrium between the Type 1 and Type 2 photoproducts that is modulated by the PHY domain and imply that effects on the 15E pK_a_ may provide a general explanation for Type 2 variants that undergo photoisomerization but fail to form P_fr_.

### The Pfr CD spectrum is associated with the protonated chromophore

**Fig. 10:**
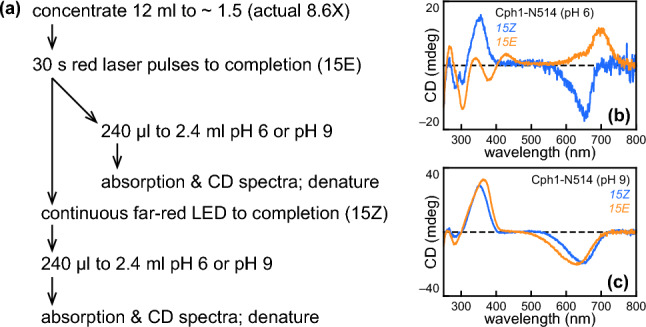
Characterization of Cph1-N514 CD spectra at pH 6 and pH 9. **a** Scheme for concentration and characterization of Cph1 is shown. **b** CD spectra are shown for Cph1-N514 at pH 6, using the color scheme of Fig. 1a. **c** CD spectra are shown for Cph1-N514 at pH 9, using the same color scheme

Our studies, thus, demonstrated the presence of a distinct Type 2 photoproduct in both absorption and CD spectroscopy and the presence of a pH-dependent equilibrium between the Type 1 and Type 2 photoproducts using absorption spectroscopy. We, therefore, sought to examine pH effects on the CD spectra. To have sufficient material for adequate CD spectra after tenfold dilution into different buffers, we were limited to characterization of wild-type N514 at pH 6 and pH 9 after concentration at pH 7.8. Parallel experiments examined the two photostates (Fig. 10A), and residual *15Z* signals were subtracted from *15E* spectra as described above. Both conditions gave rise to the expected effects. At pH 6, the *15E* CD spectrum was similar to those of Type 1 variants such as I208L or Y203F (Fig. 10B), with a simple positive band in the far-red region and a complex Soret region. At pH 9, the *15E* CD spectrum instead resembled Type 2 variants (Fig. 10C), with a simple, negative red band and a simple, positive Soret band. Comparison of the S_0_–S_1_ peak wavelengths and of the S1:S2 ratio for the absolute value of the CD spectra (to account for differences in sign; Fig. S3E) again illustrated that Type 1 variants are similar to N514 at pH 6, but that Type 2 variants instead are similar to N514 at pH 9 (Fig. S3F). These experiments directly demonstrate that the pH-dependent equilibrium between Type 1 and Type 2 photoproducts contributes to the observed CD spectrum of Pfr.

## Discussion

We conclude that the anomalous P_fr_ CD spectrum of Cph1 includes overlapping signals from at least two distinct populations: true P_fr_ and the deprotonated alternative photoproduct P_ALT_. We show that these species are in equilibrium, with modulation of the *15E* chromophore pK_a_ as a possible tuning mechanism for formation of true P_fr_. This work also builds on previous examination of P_r_ heterogeneity in Cph1 [18, 19, 21, 22, 40, 80]. Previous studies identified two *15Z* states in equilibrium: P_r_-I (or photo-P_r_) and P_r_-II (or fluor-P_r_), with P_r_-I having much higher photoactivity and the intensely fluorescent Y176H variant perturbing this equilibrium [82]. This equilibrium is also pH sensitive, with a pK_a_ of 7.5 [21]. However, the underlying event in this case does not seem to be a change in chromophore protonation state; PCB deprotonation in 15Z Cph1 is instead associated with a higher pK_a_ of approximately 9 and with formation of a species absorbing in the green to orange region of the spectrum [80]. P_r_-I is more photoactive than P_r_-II. P_r_-II has higher fluorescence, a longer excited-state lifetime, and is favored at higher temperature. This results in an inverse Arrhenius effect in time-resolved absorption spectroscopy, with slower excited-state decay at higher temperature. [19]

### Formation of P_*ALT*_ and P_fr_ during photoconversion

Our work reveals a somewhat similar equilibrium between true P_fr_ and P_ALT_. P_fr_ formation in Cph1 involves a mesoscopic transition, with the P_fr_ state behaving as a tense, high-energy protein conformation relative to P_r_ [58]. This transition is thought to occur late in the photoconversion process, after initial photoisomerization and formation of the deprotonated Meta-Rc intermediate. We propose that the Type 2 photoproduct P_ALT_ is synonymous with Meta-Rc; in other words, Meta-Rc is a photoproduct in pH-dependent equilibrium with P_fr_ rather than a true intermediate. In time-resolved absorption spectroscopy, signals assigned to the deprotonated Meta-Rc species appear after a delay associated with primary photoisomerization and evolution of the resulting Lumi-R photoproduct. The Meta-Rc signals then decrease as P_fr_ appears. However, this experiment alone cannot detect whether the supposed Meta-Rc intermediate decays irreversibly to zero, as would be expected for a true reaction intermediate. We propose that P_ALT_ (or Meta-Rc) forms first, and its concentration then drops as it equilibrates with P_fr_. This proposal is also consistent with conserved dynamics during the initial photoisomerization reaction leading to the primary photoproduct. Based on static spectra, we had previously proposed that clockwise rotation of the D-ring about the 15,16-bond was associated with formation of the α-facial *15E* D-ring in bacteriophytochromes, whereas inversion of CD in Cph1 could proceed via counter-clockwise rotation [16]. However, our current work demonstrates that P_ALT_ and P_fr_ are in equilibrium. We propose that P_ALT_ forms first and then equilibrates with P_fr_, consistent with the proposed equivalence of P_ALT_ and Meta-Rc. The observed conservation of CD in P_r_ and P_ALT_ is consistent with a conserved clockwise direction of rotation, as has been observed [83]. The initial photoproduct and P_ALT_ would then retain an α-facial D-ring, and inversion of CD only occurs upon final population of the P_fr_ state.

### Photoproduct equilibrium and the facial disposition of the 15E chromophore

The structural basis for interconversion of P_ALT_ and P_fr_ remains a question for future study. Direct deprotonation of the *15E* chromophore in P_ALT_ might facilitate D-ring motions by reducing intramolecular steric clashes, but our current data do not distinguish between this simple hypothesis and protonation/deprotonation of some other residue. The presence of multiple *15E* species in phytochromes may also complicate structural analysis of P_fr_ and attempts to elucidate the mechanism for sensing far-red light with an orange-absorbing chromophore. Photoproduct structures have recently been reported for full-length Arabidopsis phytochrome B in complex with an interacting protein at pH 7.8, with ~ 3.1 Å resolution [84], and for a PAS-GAF truncation of soy phytochrome A at pH 8.5, with ~ 2.2 Å resolution [85]. Both structures confirm the expected 5–*Z,syn* 10–*Z,syn* 15–*E,anti* chromophore geometry for the photoproduct, with poor density for the C-ring propionate side chain and with an α-facial A-ring disposition very similar to that seen in P_r_. The D-ring is also modeled as α-facial in both cases, but neither structure is able to resolve density for the C17-methyl group, which makes discrete protein–chromophore interactions important in identifying the β-facial Pfr D-ring in MAS-NMR work on the PAS-GAF-PHY truncation of oat phytochrome A3 [60]. Interestingly, the high-resolution structure shows density for the C18 side chain on both faces of the bilin [85], consistent with the presence of multiple *15E* species with distinct D-ring dispositions, but only the α-facial density was modeled. The poor resolution observed for the C-ring propionate in the recent structures is also surprising in the context of the MAS-NMR studies. MAS-NMR work identified well-resolved hydrogen bonds in the P_fr_ state between this moiety and two conserved His residues, one of which was bridged by a water molecule [60]. Our current work provides a note of caution in interpreting these structures, because we demonstrate that photoconversion of phytochromes can produce multiple species in equilibrium. Moreover, the characteristic CD signatures of P_ALT_ and P_fr_ are seen in plant phytochrome as well as in Cph1 [66]. Hence, loss of P_r_ upon red illumination would be expected to generate a mix of *15E* species in both cases based on our observations.

We propose that these structures are reporting a mix of two species equivalent to the two species seen in the current work on Cph1, with P_ALT_ matching the modeled α-facial D-ring and with true P_fr_ matching cryptic β-facial density. In this view, the final equilibrium between P_ALT_ and P_fr_ involves migration of the D-ring from the α-face to the β-face, resulting in the establishment of new protein–chromophore interactions observed in MAS-NMR and perhaps stabilizing the mesoscopic transition to the tense P_fr_ state. A subset of phytochromes from prasinophyte algae fail to exhibit inversion of CD [67]; this may reflect a different P_fr_ structure, or such phytochromes may have a red-shifted P_ALT_ species that contributes negative CD signals in the far-red region. Our interpretation implies that P_ALT_ is always present in a P_fr_ population, consistent with a true chemical equilibrium and with the presence of a complex *15E* CD spectrum in N514 even at pH 6 (Fig. 10B). Differences in absorption spectroscopy between Type 1 and Type 2 variants seen in our current work are consistent with a higher extinction coefficient for P_fr_ than for P_ALT_ (Figs. 1, S1, 3, and 4). The surprisingly robust P_fr_ formation observed *in crystallo* for the PAS-GAF truncation of soy phytochrome A [85] could reflect an altered bilin pK_a_ value for this protein, either intrinsically or in the crystal. However, efficient P_fr_ formation as assayed via absorption spectroscopy does not preclude formation of a significant amount of P_ALT_ because of this difference in extinction coefficients, as we observed in the mixed variants H202A and M267A (Fig. 6F–I).

The presence of at least two *15E* species, thus, may complicate structure determination for P_fr_. However, our analysis need not conflict with or detract from the extensive studies of Cph1 using vibrational and/or NMR methods. P_ALT_ is blue-shifted relative to P_fr_, so it would be expected to give weaker signals than P_fr_ in resonance-enhanced vibrational spectroscopies using light tuned for resonance or pre-resonance with P_fr_. In addition, true P_fr_ is associated with a rigid or tense state in NMR studies that also support a β-facial D-ring [58, 60]. Such a rigid structure may facilitate preferential detection of P_fr_ in NMR or vibrational spectroscopies relative to P_ALT_, which is associated with a broad absorption peak and hence may reflect a less ordered bilin-binding pocket that might be subject to exchange broadening. This is also consistent with the proposal that the deprotonated species in Cph1 is associated with increased protein mobility [22, 86].

### Comparison with other biliprotein photoreceptors

The α-facial D-ring density modeled in the soy phytochrome A photoproduct structure [85] led to a reinterpretation of the CD spectra based on work done with BV-utilizing bacteriophytochromes [87]. In this view, phytochromes using BV would give different responses in CD spectroscopy not because of differences in the spatial location of the D-ring, but rather because the A-ring is part of the conjugated π system in BV but not in PCB or PΦB [85]. However, this analysis neglects the A-ring amide moiety, which is in-plane with the 4,5-double bond and hence can be a component in the π system. The importance of this amide in the spectral properties of PCB can readily be seen by comparing PCB-containing biliproteins to those instead containing phycoviolobilin (PVB), in which the 4,5-bond is saturated and the A-ring amide is out of conjugation. Incorporation of either PCB or PVB into the same phycobiliprotein scaffold has been achieved using recombinant systems, with PVB resulting in a pronounced blue shift [88]. Similar effects were seen upon comparison of PΦB and its 4,5-saturated equivalent, the non-physiological phytoviolobilin [88]. These results, thus, indicate that the A-ring remains in conjugation in PCB and PΦB, albeit with one less bond, so one would expect the A-ring disposition of PCB or PΦB to have an effect on the CD spectrum. The A-ring adopts an α-facial disposition in all PAS-GAF, PAS-GAF-PHY, or full-length phytochrome structures. The D-ring and A-ring are on opposite sides of the center of pseudo-symmetry at C10, so the signs of their contributions on the same face are also opposed. Hence, an α-facial A-ring would be expected to contribute positive CD for the S_0_–S_1_ transition. PVB adducts of Cph1 or other phytochromes have not been reported, but the phycoerythrobilin (PEB) adduct of Cph1 exhibits simple positive CD on the S_0_–S_1_ band and simple negative CD on the S_0_–S_2_ band [11]. PEB has a saturated 15,16-bond, so it is not capable of photoconversion and has no conjugated D-ring [89]. The CD spectrum of the Cph1 PEB adduct, thus, is consistent with contributions from an α-facial A-ring under the assumption that the B- and C-rings remain approximately coplanar [16]. Notably, PCB, PΦB, and PEB have chemically identical A-rings, so the CD signatures of PEB in Cph1 would seem consistent with a similar contribution from the A-ring in PCB that is obscured by the stronger signals of the more strongly conjugated D-ring.

Studies of red/green CBCRs provide further support for this interpretation. In this subfamily, structures are available at atomic resolution for both photostates of Slr1393g3 (via X-ray crystallography) [78] and of NpR6012g4 (via solution NMR spectroscopy) [77]. In both cases, the photoproduct adopts a twisted configuration with an α-facial D-ring and a β-facial A-ring. In contrast to phytochromes, both rings adopt the same pseudo-helicity, so the CD signals from the A- and D-rings should reinforce each other. Signals from this photoproduct state are indeed stronger than those for P_ALT_ (Fig. 6A), consistent with this interpretation. The A-ring effect can also be estimated quantitatively by comparing the photoproduct relative rotational strength of red/green CBCRs to that of green/teal CBCRs (Fig. 6A). These two CBCR groups generate similar trapped-twist photoproducts, but green/teal CBCRs incorporate PVB rather than PCB [52, 53, 79]. Comparison of the peak wavelengths for native and denatured photoproducts implicates a similar degree of twist for the two species [79]. Relative rotational strengths for the *15E*–PCB photoproduct of red/green CBCRs (–63.7 ± 5.1 mdeg/Abs, *n* = 6) are stronger than those for the *15E*–PVB photoproduct of green/teal CBCRs (–34.3 ± 8.3 mdeg/Abs, *n* = 5), indicating that some of the observed CD signal in the red/green case is indeed arising from the A-ring. In Cph1, we propose that the weak signals of Type 1 variants would arise from internal cancelation due to the presence of P_ALT_, consistent with comparison of the CD spectra of different Type 1 variants (Fig. 6D, E). Type 2 variants have spectra dominated by P_ALT_ and hence have stronger relative rotational strengths than Type 1 variants (Fig. 6A). However, the A-ring remains on the α-face in all available structures of knotted phytochromes, so the observed relative rotational strength is weaker than that observed in the red/green photoproduct.

The photoproduct equilibrium that we report, thus, involves changes in protonation and likely involves a change in facial disposition of the D-ring. A change in chromophore protonation is consistent with the pH-dependent changes in absorption and CD spectroscopy, and our estimated pK_a_ of 8.5 for wild-type *15E* N514 is quite comparable to the value of 8.6 reported for the green/red CBCR SyCcaS in its red-absorbing 15E state [54]. A change in facial disposition of the bilin D-ring remains consistent with CD spectra and with MAS-NMR studies [16, 60]. The photoproduct equilibrium seems concomitant with refolding of the surrounding protein matrix to give the rigid or tense P_fr_ structure via a mesoscopic transition [58]. This tense conformation may in part be necessary to stabilize the far-red-absorbing configuration of the chromophore, which is not found in the absence of protein structure (Fig. 1C, D).

### What is the chemical basis for the reversed Pfr to Pr conversion under red light at low pH?

The equilibrium between multiple photoproduct species also provides an explanation for the counterintuitive “reversed” formation of P_r_ from P_fr_ with red light at low pH (Figs. 7–9). Dark reversion is slow on the timescale of these experiments (Fig. 8G), so significant conversion between the *15Z* and *15E* configurations must proceed via the forward and reverse photochemical pathways. Moreover, red light does not specifically illuminate P_r_: the P_fr_ spectrum retains some red absorption even after subtraction of residual *15Z* signals (Fig. 8D), and both species are excited by a handheld red laser pointer operating at approximately 632 nm (Fig. S4). This laser falls at approximately 70% of peak absorption for Pr and 40% for Pfr. This wavelength is also quite close to maximal absorption for the Type 2 photoproduct P_ALT_. However, the presence of forward photoconversion at pH 9 (Figs. 7F and 8F) indicates that photoconversion of P_ALT_ is inefficient in wild-type Cph1, consistent with the poor reverse photoconversion seen in Type 2 variants such as Y203A (Fig. 5C). Hence, the photoequilibrium established under red light will be largely determined by the relative absorption cross-sections and/or primary photochemical quantum yields for P_r_ and P_fr_ at these wavelengths. [90]

**Fig. 11:**
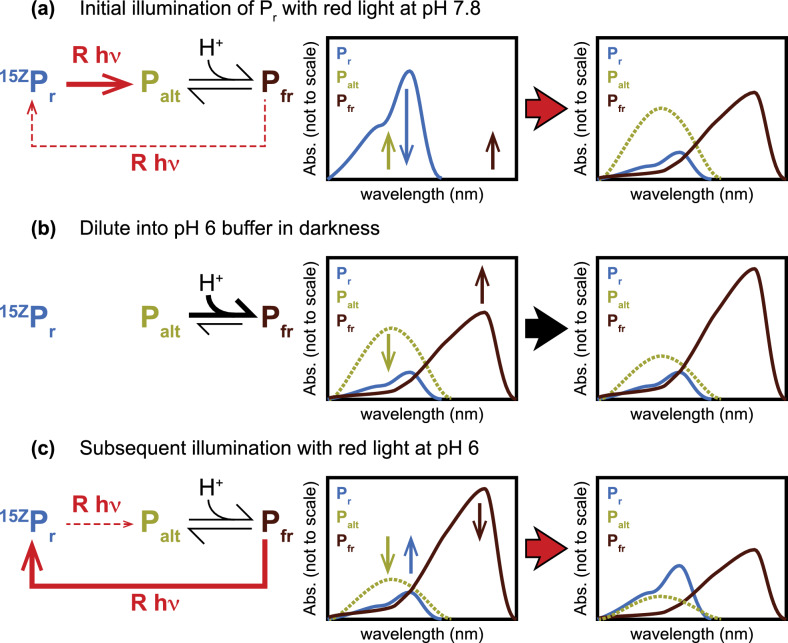
Restoration of photoequilibrium causes reversed photoconversion at pH 6. **a** Initial illumination of Pr with red light establishes a photoequilibrium due to the spectral overlap between P_r_ and P_fr_ (Fig. S4). The low quantum yield of P_ALT_ (observed in Type 2 variants and at high pH) means that its photoconversion is negligible. **b** Subsequent dilution into pH 6 buffer in darkness favors conversion of the deprotonated P_ALT_ into the protonated P_fr_, making P_fr_ in excess relative to the prior photoequilibrium. **c** Illumination with red light restores photoequilibrium, resulting in apparently reversed photoconversion as excess P_fr_ is converted to P_r_

Reversed photoconversion was observed at low pH after initial red illumination of P_r_ at pH 7.8 to generate P_fr_. Due to the spectral overlaps discussed above, initial illumination of the *15Z* state with red light at pH 7.8 establishes a photoequilibrium rather than proceeding to completion (shown as a qualitative scheme in Fig. 11A). The *15E* photoproduct is itself an ensemble of multiple species in equilibrium at pH 7.8. This ensemble is diluted into pH 6 buffer in darkness. The pH change implies that the pH-dependent equilibrium between the photoproducts is shifted (Fig. 11B). This change favors formation of the protonated P_fr_ population relative to P_ALT_, but the concentration of P_r_ is not significantly changed due to the slow dark reversion and the absence of light. Relative to the concentrations at photoequilibrium, P_r_ is, thus, unchanged, but P_fr_ is increased: the system is no longer at photoequilibrium, and P_fr_ is now in excess. Illumination of this ensemble with red light is not specific for P_r_, as noted above; rather, it favors restoration of photoequilibrium, with P_r_ and P_fr_ as the reactive species. Under conditions of excess P_fr_, restoration of photoequilibrium requires the conversion of excess P_fr_ into P_r_ and results in reversed photoconversion under red light (Fig. 11C). We, therefore, conclude that the counterintuitive reversal of the direction of photoconversion at low pH in this experiment is a consequence of the underlying equilibria we have elucidated in these studies.

### ***The P***_***fr***_*** state of Cph1 is heterogeneous***

Our work, thus, provides new insight into the P_fr_ state of Cph1. The anomalous CD spectrum of this state can be explained without reference to vibronic coupling or vibrational fine structure [68, 69]; instead, it arises via the presence of multiple species giving rise to overlapping and internally canceling spectral components. This work may also have implications for future studies of the P_fr_ state. For example, the equilibrium between P_fr_ and P_ALT_ may pose a pitfall for structural determination, as noted above. Our panel of variants also provides potentially useful reagents for future studies. The S474C variant might be a particularly interesting tool for examining the equilibrium between P_ALT_ and P_fr_. It allows effectively complete conversion to the 15E state, has a large *15E* pK_a_ shift, and retains facile reverse photoconversion at pH 7.8. This variant, thus, might allow characterization of P_ALT_ using vibrational spectroscopy or even NMR; it might also allow application of rapid-mixing techniques to lower pH and monitor P_fr_ formation from P_ALT_ on a timescale of milliseconds. In principle, it might also be possible to observe this equilibration using light-induced pH-jump strategies [91]; however, current tools for such techniques pose considerable complications due to the overlapping absorption bands of the chromophore and the available reagents.

### An unanswered issue

Lastly, it is important to note that the current work does not yet provide a full explanation of the Type 1 Pfr CD spectrum. In particular, the positive CD signals in the region of 400–450 nm (Figs. S1C and 7B) are red-shifted relative to those associated with P_ALT_ and with the observed absorption peak in this region. Therefore, there may be an additional species in this region, consistent with reports of anomalous blue fluorescence in plant phytochromes [92, 93]. Such blue-fluorescent species were proposed to arise due to formation of C10 adducts [92], a common tuning mechanism in CBCRs [94]. Such states have been characterized using absorption and CD spectroscopy in a broad range of CBCRs [5, 51, 52, 81, 95, 96], but less is known about their fluorescence properties. Future comparative studies may, thus, permit a complete description of the P_fr_ CD spectrum in this region.

## Methods

### Protein expression and purification

N514 variants with point substitutions were constructed via site-directed mutagenesis using the QuikChange system (Agilent). All Cph1 constructs and variants were expressed as intein-CBD fusion proteins in *Escherichia coli* strain LMG194 as described [16, 97], co-transforming this strain with plasmid pBAD-Cph1(N514)-CBD for Cph1 expression and plasmid pPL-PCB [98] for co-expression of the HO and PcyA enzymes required for synthesis of PCB [99]. Transformed cells were selected on rich medium (RM) or Lysogeny Broth (LB) agar plates containing 100 mg/mL ampicillin and 50 mg/mL kanamycin. Individual colonies were cultured overnight in RM or LB liquid medium containing 50 mg/mL ampicillin and 25 mg/mL kanamycin and inoculated at a 1:500 ratio (v/v) into 100 mL of RM. Upon reaching an optical density (OD) of 0.5 at 600 nm, the culture was transferred to 900 mL of LB containing 50 mg/mL ampicillin, 25 mg/mL kanamycin, and 1 mM isopropyl β-D-1-thiogalactopyranoside (IPTG). After 1 h at 37 °C with shaking at 250 rpm in a shaker-incubator (Innova series from New Brunswick Scientific), arabinose was added to a final concentration of 0.002% (w/v) followed by incubation for 1 h prior to a decrease in the temperature to 20 °C for 16 h. Cells were harvested by centrifugation at 5000 g for 10 min and stored at −80 °C. For protein preparation, a frozen pellet was resuspended at 1 g of wet weight/4 mL of lysis buffer A [25 mM HEPES-Na (pH 8.0), 500 mM NaCl, and 1 mM EDTA] and 0.1% (v/v) Triton X-100. Cells were lysed by two passes through a microfluidizer (Microfluidics M-110Y) at 15,000 psi, and all subsequent steps were conducted at 4 °C. The crude lysate was clarified by centrifugation (35,000 rpm, Ti-60 Beckman, 30 min) and applied to a 30 mL chitin column (New England Biolabs), which was washed thoroughly with buffer A at a rate of 1 mL/min. Intein-mediated cleavage was induced by flushing the column with 1 bed volume of elution buffer (buffer A + 1 mM dithiothreitol), followed by overnight incubation at 4 °C. Eluted fractions containing Cph1Δ(N514) were pooled and dialyzed overnight at 4 °C in 1 L of TKKG buffer [25 mM TES-KOH (pH 8.0), 25 mM KCl, and 10% (v/v) glycerol]. Wild-type N514 Cph1 has been expressed over five times using this procedure, without significant prep-to-prep variation. In the course of this work, the H202A and M267A variants were expressed and purified twice with consistent results, as was N322 Cph1. Other variants were characterized using a single preparation each.

The Y203A variant was also expressed as apoprotein, following the same procedures but omitting pPL-PCB and kanamycin. In vitro assembly with PCB (Frontier) used a modification of published procedures for Cph1 and CBCRs [16, 52, 57]. Reactions were performed in TKKG buffer with tris(2-carboxyethyl)phosphine hydrochloride (TCEP) added to a final concentration of 1 mM from a freshly prepared stock immediately before assembly reactions were set up. Apoprotein was present at a final volume of 1 mL, and PCB was added such that the apophytochrome was present in two-fold to four-fold excess. After incubation in darkness for 2 h, the resulting sample was characterized using absorption and CD spectroscopy. Dialysis after in vitro assembly [16, 57] was eschewed in this case due to concerns that the unidirectional photoconversion of this variant would prevent characterization of the *15Z* state in the event of an untimely light leak.

### Spectroscopy

Absorption spectra were acquired at 25 °C using Cary 50 or Cary 60 spectrophotometers equipped with a temperature-controlled cuvette holder modified to allow top-down illumination using a filtered xenon source and a liquid light guide [16, 53]. Actinic illumination with this source used band-pass filters (600 ± 20 nm, 580 ± 20 nm, 650 ± 20 nm, 720 ± 5 nm). Some samples were instead illuminated with a 728 nm LED array or with a red laser pointer (632 nm). Typical spectra were acquired at every 2 nm, with 0.125 s data acquisition per wavelength (approximately 80 s for scanning 250–900 nm). For examination under denaturing conditions, 100 µl of protein sample was added to 1 ml of 6 M guanidinium chloride/1% HCl (v/v). Denatured spectra are reported after subtraction of the same-day buffer baseline. Photoconversion of denatured samples used illumination for 1–2 min with the xenon source fitted a 320 nm long-pass filter. Difference absorption spectra were calculated as (*15Z*–*15E*). Denatured difference spectra reported in Fig. 1F were normalized by dividing by the peak observed absorbance for the red band in the *15Z* denatured spectrum, as were normalized absorption spectra. Other normalized difference spectra were normalized by dividing by the observed *15Z* peak ∆Absorbance. CD spectra were acquired using an Applied Photophysics Chirascan at 25 °C. Concentration of some samples was adjusted using Amicon Ultra 0.5 ml spin concentrators (Merck Millipore) following the manufacturer’s instructions.

### Data analysis

Gaussian fitting followed our previous procedure for CD spectra of algal phytochromes [67]. Relative rotational strength was calculated by dividing the observed strongest CD (highest positive or lowest negative value, in millidegrees) by the peak absorbance value for the same band. S1:S2 ratio for absorption spectra was calculated by dividing peak absorption of the S_0_–S_1_ transition by that for the S_0_–S_2_ transition; the equivalent ratio for CD spectra was calculated after first taking the absolute values of the spectra. The extent of photoisomerization (Table 1 and Fig. S2) was estimated by taking denatured spectra for RcaE in the completely *15E* or *15Z* states and using them to calculate a series of spectra in 5% increments, generating a standard curve [54]. The spectra in this standard curve were then normalized by dividing by the maximum value and were visually compared to similarly normalized denatured photoproduct spectra.

For pH-dependence studies, spectra at each pH value (including the buffer blank) were re-zeroed using the mean observed absorbance at 850–900 nm, and the buffer was then subtracted. Each spectrum was then normalized using the peak absorbance on the aromatic amino acid band (250–300 nm) to correct for possible differences in dilution factor. For S474C, peak chromophore absorption values from the resulting spectra were plotted against pH without further corrections, because the residual *15Z* bilin is negligible in this variant (Fig. S2). The pK_a_ was then estimated as described [54, 81] under the assumption that a single titrating group was responsible for the observed changes. For N514 and N322, the *15Z* spectrum was then scaled and subtracted from the photoproduct spectra to account for the residual *15Z* present at photoequilibrium (Fig. S2). The resulting spectra were renormalized on the UV band to correct for the different (A_bilin_/A_280_) in *15Z* and *15E* spectra, and the resulting peak chromophore absorbance values were used for nonlinear regression analysis under the same assumption.

## Conflict of interests

The authors declare no competing interests.

## Supplementary Information

Below is the link to the electronic supplementary material.

Supplementary file1 (DOCX 810 KB)

## Data Availability

Data for figures and tables are available through the DataDryad repository (URL: 10.5061/dryad.rn8pk0pp7). This includes raw and normalized absorption spectra and difference spectra and CD spectra and absorption data from dark reversion time courses. It also includes derived values used for analyses including percent photoisomerization, Gaussian fitting, relative rotational strength, and pKa values.
