# Supplementary material for: Circular dichroism spectroscopy reveals multiple phytochrome photoproducts in equilibrium
Source: Photochem Photobiol Sci. 2025 Jul 18;24(8):1351–71. doi: 10.1007/s43630-025-00763-2 (PMC12420706; doi:10.1007/s43630-025-00763-2)
Supplement: Supplementary file 1 — Supplementary file1 (DOCX 810 KB) [file 43630_2025_763_MOESM1_ESM.docx]

SUPPLEMENTAL INFORMATION for Rockwell & Lagarias, “**Circular dichroism spectroscopy reveals multiple phytochrome photoproducts in equilibrium”**


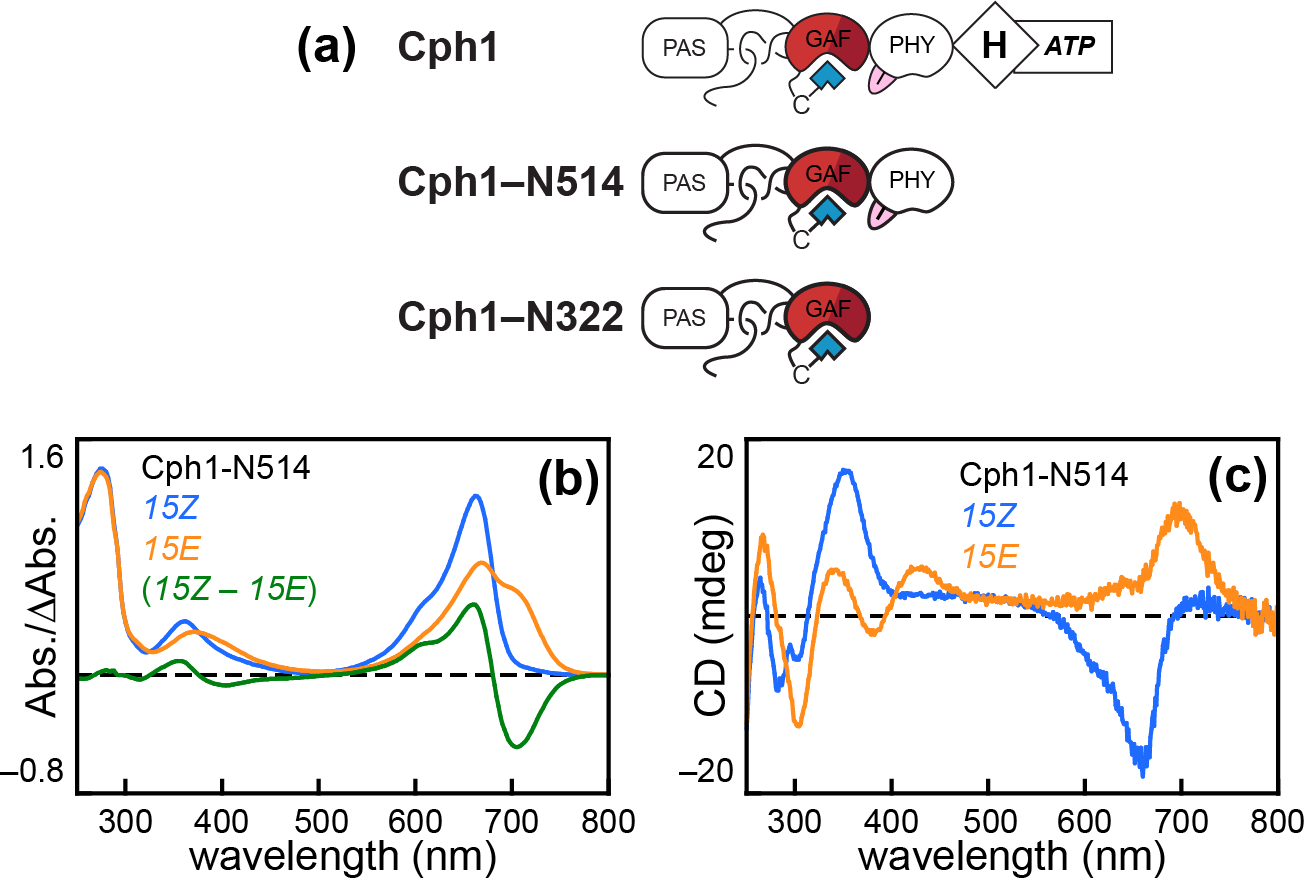


**Figure S1. Photoconversion of model cyanobacterial phytochrome Cph1.** (a) Domain architecture diagrams are shown for full-length Cph1 and for the Cph1-N514 and Cph1-322 truncations used in this work. (b) Absorption spectra are shown for Cph1-N514 in the *15Z* P_r_ (blue) and *15E* P_fr_ (orange) states, with the (*15Z* – *15E*) difference spectrum shown in green. (c) CD spectra are shown for the same sample as in panel (b), using the same color scheme. No difference CD spectrum is shown.


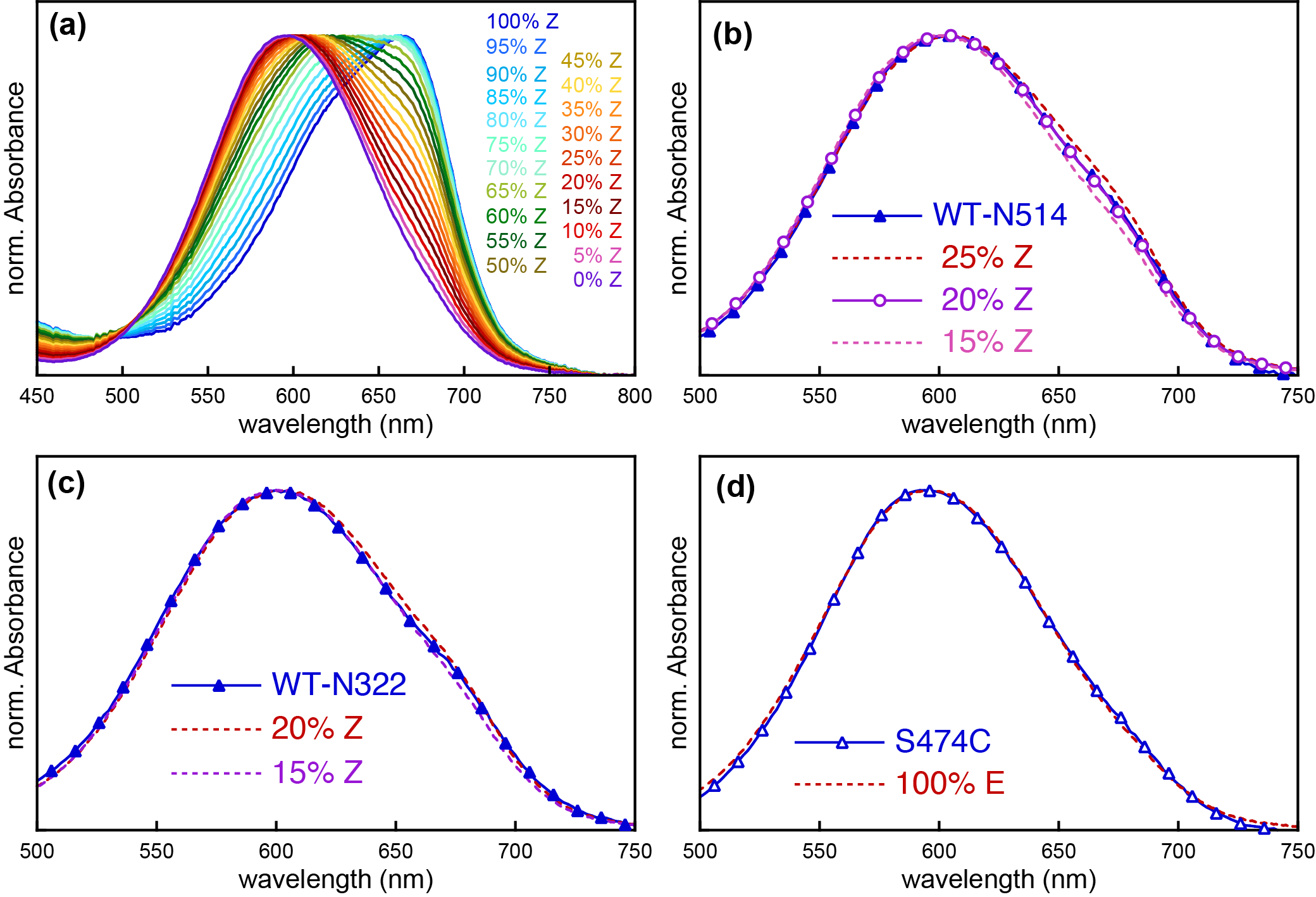


**Figure S2. Estimation of photoisomerization using an acid denaturation assay.** (a) Effectively complete *15Z* and *15E* samples of RcaE were used to calculate spectra at the indicated levels of photoconversion under acidic denaturing conditions, providing an approximate standard curve. (b) The absorption spectrum of denatured *15E* wild-type Cph1-N514 (blue line and filled triangles; spectrum from Fig. 1D) is compared to calculated reference curves for 25% *15Z*/75% *15E* (dashed red line), 15% *15Z*/85% *15E* (dashed pink line), and 20% *15Z*/80% *15E* (solid magenta line and empty circles). (c) The absorption spectrum of denatured *15E* wild-type Cph1-N322 (blue line and filled triangles; spectrum from Fig. 1C) is compared to calculated reference curves for 20% *15Z*/80% *15E* (dashed red line) and 15% *15Z*/85% *15E* (dashed magenta line). (d) The absorption spectrum of denatured *15E* S474C Cph1-N514 (blue line and filled triangles) is compared to the experimental reference for 100% *15E* (dashed red line).


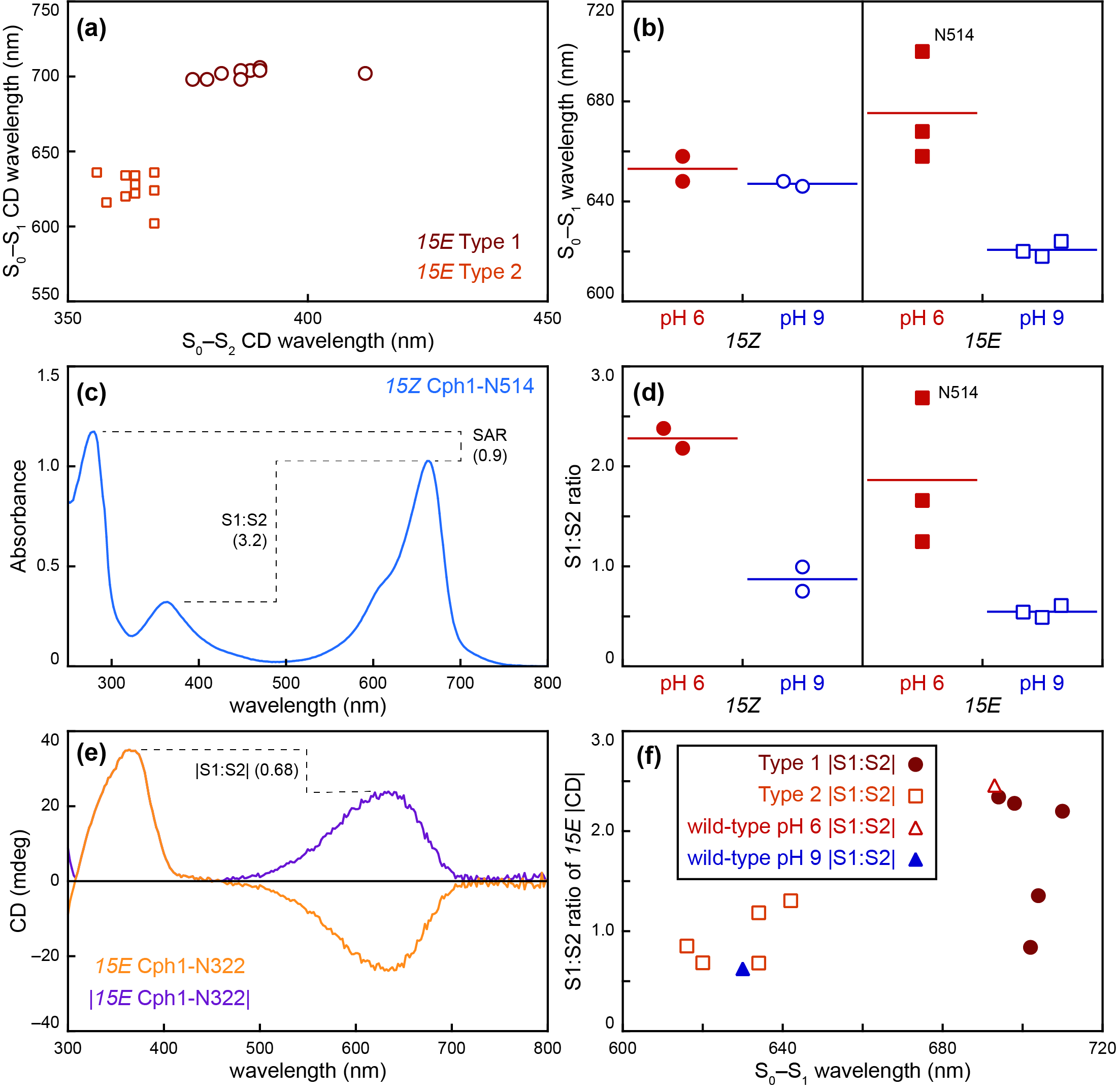


**Figure S3.** **Additional analysis of *15E* states of Cph1.** (a) Peak wavelengths for maximum observed CD (irrespective of sign) are plotted for the S_0_–S_2_ (*x-*axis) and S_0_–S_1_ (*y-*axis) transitions for Type 1 (large brick red circles) and Type 2 (small ochre squares) Cph1 variants. (b) Peak wavelengths for the S_0_–S_1_ transition are plotted for Cph1-N322 (Fig. 7), Cph1-N514 (Fig. 8), and S474C Cph1-N514 (Fig. 9) in the *15Z* (*left*, circles) and *15E* (*right*, squares) states at pH 6 (filled red symbols) and pH 9 (open blue symbols). (c) Definitions are shown for specific absorbance ratio (SAR) and for the ratio of the intensity of the S_0_–S_1_ transition to that of the S_0_–S_2_ transition (S1:S2 ratio), using the *15Z* spectrum of Cph1-N514 (Fig. S1B). (d) S1:S2 ratios are plotted for Cph1-N322 (Fig. 7), Cph1-N514 (Fig. 8), and S474C Cph1-N514 (Fig. 9) in the *15Z* (*left*, circles) and *15E* (*right*, squares) states at pH 6 (filled red symbols) and pH 9 (open blue symbols). (e) Observed CD (orange) and the absolute value of that trace (dark purple) are shown, using the *15E* spectrum of Cph1-N322 (Fig. 1B). (f) The S1:S2 ratio of the absolute value of the *15E* CD spectrum is plotted versus S_0_–S_1_ peak wavelength for Type 1 variants (Figs. S1, 3, and 5; filled brick red circles), Type 2 variants (Figs. 1, 4, and 5; open ochre squares), and for *15E* Cph1-N514 at pH 6 (open red triangle) and pH 9 (filled blue triangle). For panels (b) and (d), values for *15E* N514 are indicated to facilitate comparison.

**
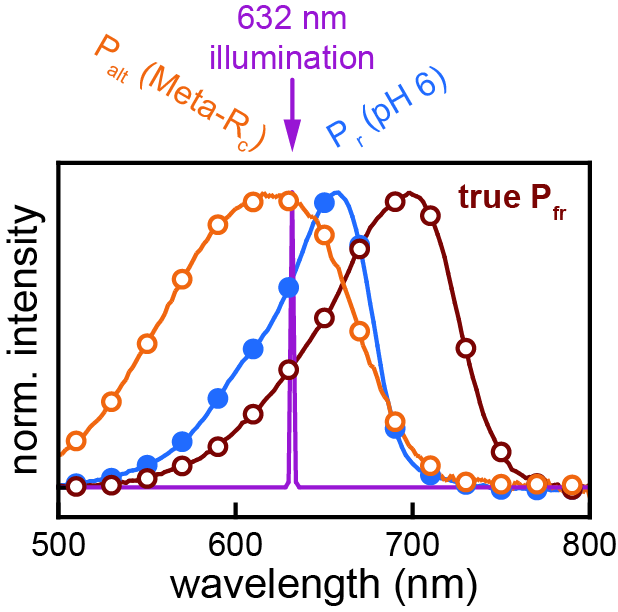
**

**Figure S4. Spectral overlap between *15Z* and *15E* states of Cph1.** Normalized absorption spectra for the *15Z* Pr state (blue line and filled circles), *15E* far-red-absorbing “true Pfr” species (brick red line and open circles), and deprotonated alternative photoproduct P_ALT_/Meta-R_c_ (dark orange line and open circles) are compared to the approximate excitation source used in pH dependence studies (632 nm laser pointer; magenta line).
